# Prevalence of suicide attempt and associations with deliberate self-harm, mental health problems, drug misuse and traumatic experiences - a cross sectional survey of the Norwegian population

**DOI:** 10.1186/s12888-024-05610-9

**Published:** 2024-02-26

**Authors:** TK Grimholt, T. Bonsaksen, T. Heir, I. Schou Bredal, L. Skogstad, Ø Ekeberg

**Affiliations:** 1 Faculty of Health, VID Specialized University, Oslo, Norway; 2 Department of Acute Medicine, Oslo University Hospital, Oslo, Norway; 3 Department of Health and Nursing Sciences, Faculty of Social and Health Sciences, Inland Norway University of Applied Sciences, Elverum, Norway; 4 Department of Health, Faculty of Health Sciences, VID Specialized University, Stavanger, Norway; 5 Norwegian Centre for Violence and Traumatic Stress Studies, Oslo, Norway; 6 Faculty of Medicine, Institute of Health and Society, University of Oslo, Oslo, Norway; 7 Faculty of Health Sciences, Department of Health and Care Sciences, UiT the Arctic University of Norway, Tromsø, Norway; 8 Psychosomatic and Consultation-Liaison psychiatry, Division of Mental Health and Addiction, Oslo University Hospital, Oslo, Norway

**Keywords:** Attempted suicide, Deliberate self-harm, Drug misuse, Mental health, Survey, Trauma

## Abstract

**Background:**

Monitoring self-reported suicide attempts (SA) with nationally representative surveys is important to initiate suicide prevention strategies. The aim of the study was to assess the prevalence of SA and compare deliberate self-harm, (DSH), mental health, drug misuse and traumas between SA and non-suicide attempters (NSA).

**Methods:**

In this cross-sectional survey of a representative sample (*N*=1757) of the Norwegian population, we compared people with self-reported SA (*n*=54) to NSA (*n*=1703) regarding sociodemographic data, mental health problems, drug misuse and exposure to trauma.

**Results:**

The prevalence of SA was 3.1 %. There was a higher proportion of welfare recipients and more deliberate self-harm, mental health problems, drug misuse and traumas in the SA group compared to NSA.

**Conclusion:**

This national study confirms the association between suicide attempt and deliberate self-harm, mental health problems, drug misuse and traumas.

## Introduction

A suicide attempt is one of the strongest predictors for suicide [1–3]. More than 700 000 people die due to suicide annually and there are many more suicide attempts for each suicide [4].

Suicidal behaviour (suicide and attempts) can be seen as a proxy measure of a population’s mental health condition, and compared to other Scandinavian countries the Norwegian suicide rates have not decreased similarly [5, 6]. Changes in the rates of suicidal behaviour and the demographic characteristics of this population have important implications for the planning of health care, and can assist more targeted initiatives for suicide prevention [4, 7]. To date, surveillance of suicide attempts has not been established in most countries and it is therefore difficult to assess epidemiological trends across time. Furthermore, since a considerable part of previous studies are based on clinical samples [8], and thus all suicide attempts are registered after treatment in the health care system [9], it is difficult to obtain reliable estimates of the prevalence in the population as a whole. Few studies have used the same methodology to estimate the lifetime prevalence of suicide attempt in the general populations, and thus comparisons are difficult. However, four cross-national studies demonstrated a range from 0.4 % to 5.9 % in the lifetime prevalence of suicide attempt [10–13].

Deliberate self-harm is more common among women in the age 15-24 years, peaks around the age of 18 years, and then decline during young adulthood [14]. Compared to the general population, people that attempt suicide more often belong to social categories associated with social destabilization and poverty [15]. From clinical populations we know that mental health problems are common in (more than 80 %) of hospitalized samples and the most frequent disorders are depression, anxiety, and alcohol misuse [16] but less is known about these associations in the general population. Further, a personal history of childhood abuse is reported risk factors for suicide attempts [17].

The aim of this study was to estimate the lifetime prevalence of suicide attempts in the general population in Norway and whether a suicide attempt was associated with deliberate self-harm, current mental health problems and drug misuse and previous traumatic experiences.

## Methods and material

This was a cross sectional survey of the adult Norwegian population. The Norwegian Central National Register selected a random representative sample (*N*=5500) of all registered Norwegian citizens => 18 years from all the 19 Norwegian counties. In the period from 2014 to 2015, they received written information about the study aims, and a comprehensive questionnaire about traumatic life experiences and general health. Further results from the study have been published elsewhere [18–20].

Of the total invited sample of 5500 persons, 9 had died, 21 could not fill out the questionnaire because of co-morbidity or old age and 499 envelopes were returned because the address was not valid. Because the survey was anonymous, we sent a reminding letter and a new questionnaire to the whole sample three times.

The final sample consisted of 1792 people (36 % response rate) and was, compared to figures from Statistics Norway, representative of the general Norwegian population in terms of age, gender, and education level (http://www.ssb.no).

### Ethics

The survey was carried out anonymously and approved by the Regional Committee for Medical and Health Research in the Southern Eastern Norway without formal written consent. The participants consented by returning the questionnaire in a sealed envelope with no personal information, and so it was not possible to identify the participants.

### The questionnaire

We registered *demographics* in age as a continuous variable, gender, educational, employment- and marital status. The participants were asked: Have you ever done any of the following: 1) Deliberately harmed yourself and 2) attempted to end your life? The answering categories were: “Never”, “Last year” and “More than one year ago”. Questions about whether they had experienced anxiety, depression, sleeping problems, eating disorders and psychoses (e.g., hallucinations) were listed with the answering categories: “No”, “Yes previously”, “Yes last month”. Further, the participants were asked about their use of alcohol, cannabis/marihuana, sedatives (diazepam), strong pain killers (codeine), opiates (heroin), and stimulating drugs (amphetamine, cocaine). The answering categories were: “No”, “Sometimes”, “Weekly”, “Daily” and “Several times daily”. *Traumatic lifetime experiences were framed as have you ever experienced the following:* Sexual abuse/ assault (rape, attempted rape or forced sexual activity).

Violence (beaten, kicked, attacked). Nature disaster (e.g., Hurricane, earthquake). With the answering categories “It happened to me”, “I witnessed it”, “I was told by a close person”, “Part of my work”, “Not sure”, “Does not apply to me”. Exposure to each of the listed experiences was classified for participants indicating ‘it happened to me’.

### Statistical analyses

Cross tabulations with chi-square and fishers exact test were used to present the proportions of suicide attempt vs. non suicide attempt according to sociodemographic variables, DSH, mental health problems and drug misuse. In the analysis of traumatic life events the category “It happened to me” was used to cross tabulate with suicide attempt and calculate the Odds ratios. The significance level was set at 5 %. SPSS., Chic. Ill version 26 was used to analyse the data.

## Results

Of all respondents (*N*=1792), *n*= 1757 (98%) answered the question about suicide attempt (SA). The lifetime prevalence of SA was 3.1 (*n*=54) % and of these 75.9 % (*n*=41) were women. There was significant difference between SA (48.2 yrs.) and NSA (53.2 yrs.) on mean age (*p*=0.03).

There was a higher proportion of welfare recipients in the SA group (23 % vs. 6 %, *p*< 0.001). Apart from this, there were no sociodemographic differences between participants with and without suicide attempt (Table 1).

**Table 1:** Sociodemographic characteristics attempted suicide vs. never attempted suicide

	**Previous suicide attempt**	**Never attempted suicide**	***p*-value**
N (1757)			
Total	**3.1 % (54)**	**96.9 % (1703)**	
**Gender**			
Female	75.9 %	52.4 %	
Male	24.1%	47.6 %	0.001
**Educational level**			
Elementary school	11.3 %	7.6 %	
High school	49.1 %	32.2 %	
College/ University	39.6 %	54.2 %	0.10
**Employment status**			
Employed, student, military	51.9 %	67.5 %	
Unemployed	1.9 %	1.3 %	
Welfare recipient	23.1 %	5.7 %	
Retired	23.1 %	25.4 %	0.000
**Marital status**			
Single	13.2 %	12.9 %	
Married/cohabiting	66 %	77.9 %	
Separated/divorced	13.2 %	5.1 %	
Widowed	7.5 %	4.1 %	0.035

*P* values from Chi square and Fischer’s exact test

There was a significantly higher proportion self-reported DSH and mental health problems in the SA group. Compared with participants without SA, the most common mental health problems were sleeping disorders last month/previously (81.6.%) and depression (84.9 %) (Table 2).

**Table 2:** Proportion of DSH and mental health problems attempted suicide vs. never attempted suicide

	**Attempted suicide *N*=54**	**Never attempted suicide *N*=1703**	***p*-value**
**Deliberate self-harm**			
Last year	10.4 %	1.9 %	
Yes previously	37.5 %	0.5 %	
No	52.1 %	97.6 %	0.000
**Depression**			
Previous month	39.6 %	7.2 %	
Yes previously	45.3 %	20.6 %	
No	15.1 %	72.1 %	0.000
**Anxiety**			
Previous month	30.8 %	6.1 %	
Yes previously	40.4 % (	14.0 %	
No	28.8 %	79.8 %	0.000
**Sleeping disturbance**			
Previous month	55.1 %	20.7 %	
Yes previously	26.5 %	15.5 %	
No	18.4 %	63.8 %	0.000
**Eating disorders**			
Previous month	25.0 %	2.3 %	
Yes previously	16.7 %	4.5 %	
No	58.3 %	93.2 %	0.000
**Psychosis (e.g.Hallucinations)**			
Previous month	0.2 %	0.1 %	
Yes previously	16.7 %	1.3 %	
No	81.3 %	98.6 %	0.000

Self-reported drug misuse (indicated as ‘sometimes’ or more often used) was significantly higher in the SA group for cannabis (26 % vs. 7.4 %), sedatives (34.7 % vs. 7.6%), strong pain killers (57.4 % vs. 33.2 %) and opiates (12.2 % vs. 2.2%) (Table 3).

**Table 3:** Proportions of Alcohol and drug misuse attempted suicide vs. never attempted suicide

	**Attempted suicide (*n*=54)**	**Never attempted suicide (*n*=1703)**	***p*-value**
**Alcohol**			
Never	17. 0%	16. 1 %	
Sometimes	43.4 %	47.6 %	
Weekly or Daily	39.6 %	36.3 %	0.83
**Cannabis/ Marihuana**			
Never	74 %	92.5 %	
Sometimes	26.0 %	6.6 %	
Weekly or Daily	0.0 %	0.8 %	0.000
**Sedatives (e.g diazepam)**			
Never	65.3 %	92.4 %	
Sometimes	26.5 %	6.6 %	
Weekly or Daily	8.2 %	1.0 %	0.000
**Strong pain killers (e.g. codeine)**			
Never	42. 6 %	66.8 %	
Sometimes	38.9 %	30.1 %	
Weekly or Daily	18.5 %	3.1 %	0.000
**Opiates (Heroine, Morphine)**			
Never	87.8 %	97.8 %	
Sometimes	12.2 %	1.8 %	
Weekly or Daily	0.0 %	0.4 %	0.000
**Stimulant drugs (Cocaine, amphetamine)**			
Never	93.9 %	93.9 %	
Sometimes	6.1 %	2.2 %	
Weekly or Daily	0.0 %	0.1 %	0.20

As displayed in Table 4 there were 33 % (*n*=18) in the SA group that reported sexual abuse (OR 9.4), 35 % (*n*=19) reported exposure to violence (OR 3.0).

**Table 4:** Traumatic experiences and suicide attempt

	**Attempted suicide**		**0R**	***P*-value**
	Yes	No		
**Sexually abused**				
Yes	33 % (18)	5 % (86)		
No	67 % (36)	95 % (1617)	9.4 (5.1-17.2)	0.000
**Exposed to violence**				
Yes	35 % (19)	15 % (260)		
No	65 % (35)	85 % (1443)	3.0 (1.7-5.3)	0.000
**Nature disaster**				
Yes	7 % (4)	8 % (128)		
No	93 % (50)	93 % (1575	0.98 (0.4-2.8)	0.976

Missing cases: *n*=80 (4.4 %)

Percentages are presented without decimals, and therefore the total per cent sometimes exceeds 100

## Discussion

The lifetime prevalence of self-reported suicide attempt was 3.1 % and three out of four who had attempted suicide were women. There was significantly more deliberate self-harm, mental health problems and use of drugs in the suicide attempt group. The odds for attempting suicide were high in association with human inflicted traumas, such as sexual abuse and violence, compared to natural disasters.

In a prevalence study from 1999 the lifetime prevalence of suicide attempt ranged from 0.72 % in Beirut up to 5.93 % in Puerto Rico [10].

Data from the World Health Organization (WHO) World Mental Health (WMH) Survey Initiative in 17 countries showed that the global cross-national lifetime prevalence of suicide attempts was 2.7% and thus somewhat lower than the prevalence in Norway. However there was a substantial variability in the prevalence of suicide attempts cross-nationally ranging from e.g., 0.5 % in Italy, 1.5 % Spain, 1.7 % Germany, 2.3 % Netherlands, 2.5 % Belgium, 3.4 % France, and up to 5.0 % in USA [11].

In a study comparing France and Spain the prevalence of lifetime suicide attempt was 3.4% in France (1.1% men, 5.4% women) and 1.5% in Spain (1.2% men, 1.7% women), with a significantly greater gender difference in France [21].

In a recent study from Belgium, the lifetime-prevalence of suicide attempts seemed to have had an increase from 2.5 % to 6.5%. Prevalence rates were higher in younger people and individuals with a primary educational level and with financial distress [22].

Previous studies have found consistent cross-national risk factors for suicide attempt as being female, younger, less educated, and unmarried [11].

Our results align with these results, as both being female and younger were associated with a higher likelihood of having attempted suicide during the lifetime. Not being married was also supported as a risk factor in our study, with a significantly higher proportion of single, unmarried, divorced and widowed in the SA group.

Mental health problems are also consistent cross national risk factors, and our findings of significantly more suicide attempts across all groups of self-reported psychiatric problems support these findings [11].

The differences between the study groups in use of drugs, might partly be explained by the high prevalence of psychiatric diagnoses and a corresponding use of medication in patients attempting suicide [16]. A strong association between physical and sexual abuse in childhood and later suicide attempt was demonstrated by Brown et.al [23], and the results in our study support these findings.

Treatment for PTSD following sexual assault in the form of prolonged exposure therapy in adolescents have been found to decrease suicide ideation [24]. However, although this is helpful, primary prevention measures are also necessary to avoid sexual abuse, violence, and exposure to other forms of potentially traumatic events among children and adolescents. The association between suicide attempts and childhood maltreatment in form of physical, sexual, and/or emotional abuse is documented in a general population study from USA where 2% of previously exposed adults reported a suicide attempt [25]. In contrast, exposure in adulthood was not associated with recent suicidality. In a study of adult outpatients with trauma, Guina et al. found a correlation between PTSD symptoms and the prevalence of suicide attempts. In this population, attempted suicide was significantly correlated with substance related problems in general and alcohol specifically [26]. This is in contrast with our study, where alcohol was the only substance not associated with SA, while the use of benzodiazepines and narcotic substances were all significantly higher in the SA group.

### Limitations

There are some limitations in this study. First, the low response rate weakens the external validity and generalization of the results. We have no information about the non-responders and therefore no possibility to analyse dropouts apart from that there were no significant differences in age, gender proportions and place of living (rural/ urban) between responders and non-responders and thus minimal biases due to socioeconomic and cultural influences. Compared to figures obtained from Statistics Norway, the proportion in active work was 61% in the current sample compared to 67% in the general population and there were 17% who lived alone in both groups. There were however, 1.3% without work and 53% with College or University education in the study group compared to general population, 4.4% and 41% respectively (http://www.ssb.no).

We therefore consider our findings to be representative of the Norwegian population.

Second, we were not able to obtain information about severity of the suicide attempts or whether the respondents had attempted multiple times. Due to the missing information of the timeline of incidents, we were not able to identify any causality between mental health drug abuse, exposures and suicide attempts. Third, the validity of retrospective reports by the adults about their adverse experiences might to some extent be biased due to recall bias.

Last, the questions and formulations employed in surveys will influence the respondent’s interpretations and therefore also the prevalence estimates. E.g., in studies of non-suicidal injury, use of dichotomous questions reduced the numbers to almost half (12.5 %) compared to use of detailed check lists (25.6 %) [27].

In a review of suicidal behaviour among adolescents, the authors found that the rates varied based on the definitions and the term suicide attempt yielded lower responses (9.7%) compared to deliberate self-harm (13.2%) [28].

Although we used both the terms suicide attempt and deliberate self-harm in our study, and could combine the overlapping groups, these limitations should be borne in mind when interpreting the results.

The questions about mental health problems are based on the respondents subjective perception and not standardized checklists.

It is therefore not reliable in terms of diagnostic criteria and the possibility to discriminate between psychiatric disorders or other well-known factors associated with suicidal behaviour [13].

### Strengths

The use of an anonymous questionnaire might increase the reliability of self-report about sensitive topics such as suicidal behaviour, as demonstrated by Evans and colleagues [28]. Further, compared to prevalence figures from registry data, clinical studies, and regional health surveys this was a national population survey that enabled us to identify people that are not necessarily treated and registered with codes for reimbursement purposes within the health care services.

### Further research

Common methodology in terms of similar use of terminology, repeated cross-sectional studies and multinational cooperation is needed to establish updated and comparable prevalence rates. It is important to study whether the prevalence of suicide attempts increases, or whether sociodemographic patterns related to suicide attempts change over time.

### Implications for suicide prevention

Suicide prevention has been a health priority in many countries, including Norway. To reduce the rates of suicide attempts primary prevention measures are necessary. This paper underlines the need to identify and hinder risk factors and exposures, especially sexual abuse.

## Conclusions

This national study confirms the associations between suicide attempt and deliberate self-harm, mental health problems, drug misuse and human inflicted traumas.

## Data Availability

The datasets used and/or analyzed during the current study are available from the corresponding author on reasonable request.
